# Characterization of the rainbow trout (*Oncorhynchus mykiss*) mucosal glycosphingolipid repertoire and *Aeromonas salmonicida* binding to neutral glycosphingolipids

**DOI:** 10.1093/glycob/cwae055

**Published:** 2024-08-07

**Authors:** John Benktander, Henrik Sundh, Kristina Sundell, Sinan Sharba, Susann Teneberg, Sara K Lindén

**Affiliations:** Department of Medical Biochemistry and Cell Biology, Institute of Biomedicine, Sahlgrenska Academy, University of Gothenburg, Box 440, Medicinaregatan 9C, Gothenburg 405 30, Sweden; Department of Biological and Environmental Sciences, Box 463, Medicinareg 7B, University of Gothenburg, Gothenburg 405 30, Sweden; Department of Biological and Environmental Sciences, Box 463, Medicinareg 7B, University of Gothenburg, Gothenburg 405 30, Sweden; Department of Medical Biochemistry and Cell Biology, Institute of Biomedicine, Sahlgrenska Academy, University of Gothenburg, Box 440, Medicinaregatan 9C, Gothenburg 405 30, Sweden; Department of Medical Biochemistry and Cell Biology, Institute of Biomedicine, Sahlgrenska Academy, University of Gothenburg, Box 440, Medicinaregatan 9C, Gothenburg 405 30, Sweden; Department of Medical Biochemistry and Cell Biology, Institute of Biomedicine, Sahlgrenska Academy, University of Gothenburg, Box 440, Medicinaregatan 9C, Gothenburg 405 30, Sweden

**Keywords:** Aeromonas salmonicida, bacterial adhesion, glycosphingolipids, rainbow trout

## Abstract

Infections pose a challenge for the fast growing aquaculture sector. Glycosphingolipids are cell membrane components that pathogens utilize for attachment to the host to initiate infection. Here, we characterized rainbow trout glycosphingolipids from five mucosal tissues using mass spectrometry and nuclear magnetic resonance and investigated binding of radiolabeled *Aeromonas salmonicida* to the glycosphingolipids on thin-layer chromatograms. 12 neutral and 14 acidic glycosphingolipids were identified. The glycosphingolipids isolated from the stomach and intestine were mainly neutral, whereas glycosphingolipids isolated from the skin, gills and pyloric caeca were largely acidic. Many of the acidic structures were poly-sialylated with shorter glycan structures in the skin compared to the other tissues. The sialic acids found were Neu5Ac and Neu5Gc. Most of the glycosphingolipids had isoglobo and ganglio core chains, or a combination of these. The epitopes on the rainbow trout glycosphingolipid glycans differed between epithelial sites leading to differences in pathogen binding. A major terminal epitope was fucose, that occurred attached to GalNAc in a α1-3 linkage but also in the form of HexNAc-(Fuc-)HexNAc-R. *A. salmonicida* were shown to bind to neutral glycosphingolipids from the gill and intestine. This study is the first to do a comprehensive investigation of the rainbow trout glycosphingolipids and analyze binding of *A. salmonicida* to glycosphingolipids. The structural information paves the way for identification of ways of interfering in pathogen colonization processes to protect against infections in aquaculture and contributes towards understanding *A. salmonicida* infection mechanisms.

## Introduction

Rainbow trout (*O. mykiss*) is a ray-finned fish of the salmonid family native to fresh and brackish water in the cold water North Pacific regions of North America and Asia. Rainbow trout is commonly grown in aquaculture also in other regions such as Europe and South America.

In fish, as in other vertebrates, the epithelia are covered by a continuously secreted mucus layer in which mucins are the main components (Linden et al. 2008). The highly glycosylated mucins have a protective functions by acting as decoys to limit direct attachment of pathogens to the epithelial cell membrane (Linden et al. 2008; Lindén et al. 2009). However, pathogens that get past the mucus barrier can potentially use the attachment to the glycosphingolipids (GSL) and other membrane bound components to facilitate infection of the host (Benktander et al. 2012; Benktander et al. 2013; Padra et al. 2018). In addition to being important determinants for host-pathogen interactions, GSL also provide an avenue for investigating pathogen adhesion specificity (Hanada 2005; Aspholm et al. 2006; Padra et al. 2019a; Bereznicka et al. 2022).

GSL from fish have not been widely researched, but a comprehensive study has been performed on Atlantic salmon epithelia (Benktander et al. 2022). In that study, many structures with ganglio and isoglobo core chains were detected, and several structures with terminal Fuc–HexNAc- and HexNAc-(Fuc-)HexNAc- were found (Benktander et al. 2022). In addition, several ganglioside structures and GSL with terminal Fucα1-3GalNAc- were found in zebrafish (*Danio rerio*) (Yamakawa et al. 2018). In rainbow trout liver GSL, four neutral GSL, glucosylceramide, galactosylceramide, globoside and lactosylceramide were found in conjunction with five acidic GSL: sulfatide, and the gangliosides GM3, GM2, GD1a and 9-O-Acetyl GD3 (Ostrander et al. 1991).

*A. salmonicida*, the etiological agent of furunculosis disease, is a bacterium associated with high morbidity and mortality in aquaculture. In rainbow trout, *A. salmonicida* was observed on gills and fins 2–4 h after an in vivo bath challenge, whereas binding to skin was absent (Bartkova et al. 2017). Further, *A. salmonicida* can utilize the intestine as a translocation route (Jutfelt et al. 2006; Jutfelt et al. 2008) and could be found in the intestine 24 h after a bath challenge (Bartkova et al. 2017). *A. salmonicida* binds to sialic acids on mucins from the Atlantic salmon mucus layer (Padra et al. 2014). The binding is affected by Ca^+2^ levels and fluid velocity. Furthermore, the growth of the bacterium is affected by *N*-acetylhexosamines (Padra et al. 2017; Padra et al. 2019b). The mucus niche is constantly renewed and thereby a relatively unstable niche for a microbe to inhabit. Adhesion to GSL provides an intimate contact to the host, and many bacterial pathogens carry adhesins that recognize GSL (Aspholm-Hurtig et al. 2004; Benktander et al. 2012; Benktander et al. 2013; Padra et al. 2018). It is not known if *A. salmonicida* can bind to rainbow trout GSL.

In this study, we investigate the composition of GSL in epithelia from rainbow trout and examined the binding of *A. salmonicida* to rainbow trout GSL.

## Results

### Yield of GSL from rainbow trout tissues

GSL were isolated from tissues pooled from three rainbow trout. The majority of GSL isolated from the stomach and intestine were neutral, whereas the majority of GSL isolated from the skin, gills and pyloric ceaca were acidic (Table 1).

**Table 1:** Dry weights of tissues and amounts of extracted GSLs from tissues pooled from three rainbow trout.

**Tissue**	**Dry weight (g)**	**Neutral GSL (mg)**	**Acidic GSL (mg)**	**Neutral GSLs/tissue (mg/g)**	**Acid GSLs/tissue (mg/g)**
Skin	13.06	14.4	29.0	1.1	2.2
Gills	4.61	9.2	23.7	2.0	5.1
Esophagus + Stomach	3.84	17.2	11.6	4.5	3.0
Pyloric caeca	11.58	42.0	243.3	3.6	21.0
Intestine	1.51	7.8	5.0	5.2	3.3

### Characterization of GSL from rainbow trout tissues

**Fig. 1:**
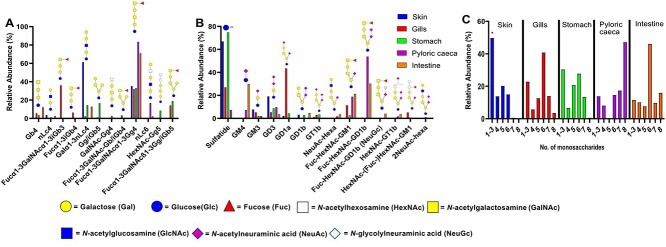
Summary of GSL found by LC–MS in rainbow trout tissues. See Table 2 for GSL structures. A) Neutral oligosaccharides derived from hydrolyzed GSL. Note that no oligosaccharides under two monosaccharides can be detected. B) Acid GSL analyzed in native state C) Estimated relative abundance of structures composed of one to eight monosaccharides in the different tissues. ^*^The relative abundance of structures with one and two monosaccharides is underestimated since the LC–MS of the neutral glycans could not detect structures smaller in size than three monosaccharides.

After isolation of GSL from the rainbow trout tissues, the GSL were characterized by LC–MS. A total of 12 neutral and 14 acidic GSL were detected (Fig. 1A and B). An estimation of the total relative abundance of each GSL were made by using the ratio of neutral to acidic GSL. Structural characterization indicated that GSL were mainly composed of isoglobo and ganglio core chains, and even a combination of these. Fucose was attached to HexNAcs in two forms; as Fuc-HexNAc-R and HexNAc-(Fuc-)HexNAc-R. The sialic acid was mainly Neu5Ac, with small amounts of Neu5Gc. Several of the acidic GSL were poly-sialylated.

### GSL from rainbow trout skin have high amounts of neolacto core

The differences in GSL expression between the tissues of rainbow trout were investigated. This demonstrated that skin had shorter glycan structures compared to the other tissues analyzed as well as a difference in core chain structures, with skin lacking globo (Gb) and isoglobo (iGb) core chains, while the other tissues contained these structures (Fig. 1A–C). Skin also differed by having a high relative abundance of neolacto based structures (38%), while the rest of the tissues had <10% relative abundance of these structures. The gills and stomach were the only tissues with GSLs containing the hybrid isoglobo/ganglio (iGb/Gg) core chains, e.g. the structures at *m/z* 868 and 1217 (Table 2).

**Table 2:** Tentative structures of the neutral and acid GSL from rainbow trout. No *m/z* of the acid structures was assigned, due to multiple *m/z* caused by different ceramides.

***m/z***	**Common name**	**Tentative structure**
706.2	Globo tetra (Gb4)	GalNAcβ3Galα4Galβ4Glcβ1Cer
706.2	Neolacto tetra (nLc4)	Galβ4GlcNAcβ3Galβ4Glcβ1Cer
852.3	Fucα3GalNAcα3-iGb3	Fucα3GalNAcα3Galα3Galβ4Glcβ1Cer
852.3	Fucα3-iGb4	Fucα3GalNAc3Galα3Galβ4Glcβ1Cer
868.2	Gal-nLc4	Galα3Galβ4GlcNAcβ3Galβ4Glcβ1Cer
868.2	Hybrid Gg/iGb5	Galβ3GalNAcβ4(Galα3)Galβ4Glcβ1Cer
909.2	HexNAc-Gg4	GalNAc3Gal3GalNAcβ4Galβ4Glcβ1Cer
1014.2	Fucα3- hybrid Gb/iGb5	Fucα3GalNAc3Galα4(Galα3)Galβ4Glcβ1Cer
1055.3	Fucα3GalNAcα3-Gg4	Fucα3GalNAc3Gal3GalNAcβ4Galβ4Glcβ1Cer
1071.3	nLc6	Gal-HexNAcβ3Galβ4GlcNAcβ3Galβ4Glcβ1Cer
1112.3	HexNAc-HexNAc-Gg4	HexNAc-HexNAc-Gal3GalNAcβ4Galβ4Glcβ1Cer
1217.3	Fucα3GalNAcα3 hybrid Gg/iGb5	Fucα3GalNAcα3Galα3GalNAcβ4(Galα3)Galβ4Glcβ1Cer
-	Sulfatide	SO_3_-Galβ1Cer
-	GM4	Neu5Acα3Galβ1Cer
-	GM3	Neu5Acα3Galβ4Glcβ1Cer
-	GD3	Neu5Acα8Neu5Acα3Galβ4Glcβ1Cer
-	GD1a	Neu5Acα3Galβ3GalNAcβ4(Neu5Acα3)Galβ4Glcβ1Cer
-	GD1b	Galβ3GalNAcβ4(Neu5Acα8Neu5Acα3)Galβ4Glcβ1Cer
-	GT1b	Neu5Acα3Galβ3GalNAcβ4(Neu5Acα8Neu5Acα3)Galβ4Glcβ1Cer
-	NeuAc-Hexa	Galβ3GlcNAcβ3Galβ3GalNAcβ4(Neu5Acα3)Galβ4Glcβ1Cer
-	Fuc-HexNAc-GM1	Fucα3GalNAc3Galβ3GalNAcβ4(Neu5Acα3)Galβ4Glcβ1Cer
-	Fuc-HexNAc-GD1b	Fucα3GalNAc3Galβ3GalNAcβ4(Neu5Acα8Neu5Acα3)Galβ4Glcβ1Cer
-	Fuc-HexNAc-GD1b (Neu5Gc)	Fucα3GalNA3Galβ3GalNcAcβ4(Neu5Gcα8Neu5Acα3)Galβ4Glcβ1Cer
-	HexNAc-GT1b	Neu5Ac{GalNAc3Galβ3GalNAcβ4(Neu5Acα8Neu5Acα3)Galβ4Glcβ1Cer
-	HexNAc-(Fuc-)HexNAc-GM1	HexNAc(Fuc-)HexNAc-Galβ3GalNAcβ4(Neu5Acα3)Galβ4Glcβ1Cer
-	Neu5Ac_2_-hexa	Galβ3GlcNAcβ3Galβ3GalNAcβ4(Neu5Acα8Neu5Acα3)Galβ4Glcβ1Cer

### Terminal epitopes of rainbow trout GSLs differ between epithelia

**Fig. 2:**
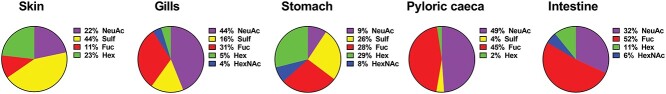
Estimated proportion of terminal moieties of the GSLs in different rainbow trout epithelia. The neutral and acidic GSL results were combined based on the weight of the fractions to obtain an estimate of the proportion of the GSL of the different types in the tissue. However, due to methodological differences, the numbers should be viewed as an estimation and not an exact result.

**Fig. 3:**
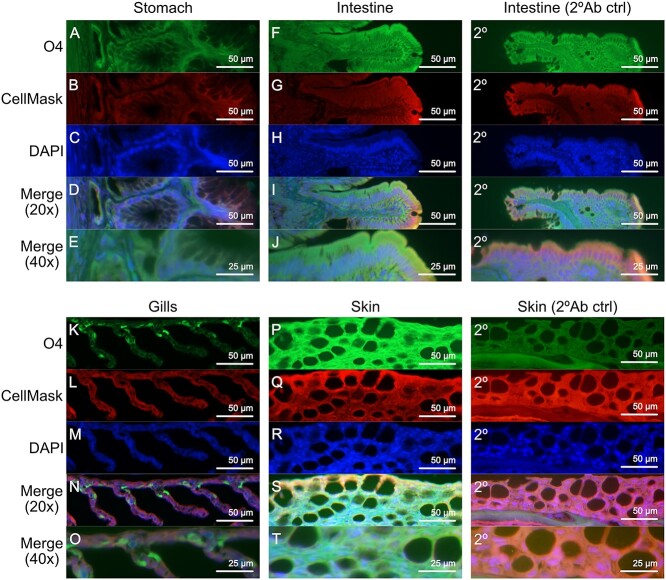
Sulfatides in stomach, intestine, gills and skin of rainbow trout. A, F, K and P) Sulfatide localization as detected with the O4 antibody (green). B, G, L and Q) Tissue outlined by CellMask (red). C, H, M and R) DAPI nuclear stain (blue). D, I, N and S) Merged channels at 20×. E, J, O and T) merged channels at 40×. (2°) Intestine and skin tissue without O4 antibody stain were used as secondary antibody background control.

The non-reducing terminal ends of the rainbow trout GSL of the different epithelia were examined. Fucose as a terminal moiety was most abundant in the intestine and pyloric caeca, but also prevalent in the gills and stomach/esophagus (Fig. 2). Sialic acid was abundant in the pyloric caeca and gill, and the highest abundance of sulfation was found in the skin (Fig. 2). Sulfatide staining of tissue sections also showed that sulfatides were most prevalent in the skin (Fig. 3). In all five tissues, sulfatides were present in cell types in direct contact with the external environment. In the gills, the sulfatide stain was only present in a low proportion of the cells (which may be chloride cells), whereas in the other tissues, the sulfatide stain was present in the majority of the cells in the surface epithelium.

### *A. Salmonicida* binds to GSL from rainbow trout gill

**Fig. 4:**
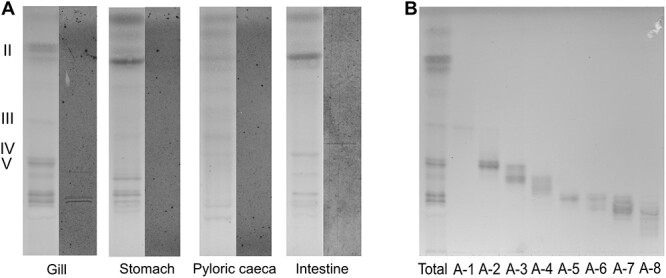
A) Binding of *A. salmonicida* to the neutral GSL from rainbow trout tissues. Roman numerals display approximate monosaccharide chain length of GSL bands. For each epithelial site: *Left*) chemical detection by anisaldehyde staining. *Right*) thin-layer chromatogram incubated with ^35^S-methionine labeled *A. salmonicida*. B) Chemical detection by anisaldehyde staining of GSL subfractions from rainbow trout gills. Total signifies the neutral GSL before sub fractionation.

Next, binding of *A. salmonicida* to the total neutral and acidic fractions from rainbow trout tissues was examined. Thereby a distinct binding of *A. salmonicida* to three neutral GSL from the gills (approx. R_f:_ 0.26, 0.27 and 0.38) and one from the intestine were identified (approx. R_f:_ 0.40) Fig. 4A). No binding to any of the acidic GSL was detected, although the assay was repeated three times (data not shown).

**Table 3:** Chemical shift data (ppm) from 700 MHz proton NMR of rainbow trout gill GSL and reference GSL, obtained in DMSO-d_6_/D_2_O (98∶2, by volume) at 30 °C. Numbers in parentheses shows the *J*-coupling constants.

		**VIII**	**VII**	**VI**	**V**	**IV**	**III**	**II**	**I**	
A-2					Fucα3	GalNAcα3	Galα3	Galβ4	Glcβ	Cer
	H1				4.74 (~2.6)	5.05(<3)	4.97(<3)	~4.28(8.8)	4.17 (~9)	
	H2				3.43	3.97	3.60	3.36	3.03	
	H3				3.61	ND	ND	3.44	3.36	
A-5			Fucα3	GalNAcα3	Galα1-3	GalNAcβ4	[Galα3]	Galβ4	Glcβ	Cer
	H1		4.73 (3.9)	5.05(<3)	4.87(<3)	4.65 (8.4)	4.92(<3)	~4.28 (~8)	4.17 (8)	
	H2		3.45	3.91	3.74	3.89	3.78	3.45	3.05	
	H3		ND	3.51	3.62	3.52	3.62	3.51	3.36	
	H4		ND	ND	ND	3.78	ND	ND	3.30	
	H5		4.05	ND	ND	3.62	ND	ND	3.27	
References										
(Ostrander et al. 1988)		Fucα3	GalNAcβ3	Galα3	Galβ4	GlcNAcβ3	[GalNAcβ4]	Galβ4	Glcβ	Cer
	H1	~4.73	4.66	4.88	4.32	4.50	4.66	4.24	4.19	
(Niimura et al. 1999)			Fucα3	GalNAcβ3	Galβ3	GalNAcβ4	[NeuAcα3]	Glcβ	Glcβ	Cer
	H1		4.76	4.66	4.29	4.87	–	4.28	4.16	
	H2		3.44	3.91	3.46	3.95		3.17	3.05	
	H3		3.60	3.52	3.49	3.55		3.77	3.35	
	H4		3.46	3.76	3.82	3.76		3.96	3.30	
	H5		4.05	3.66	3.40	3.65		3.48	3.28	
(Clausen et al. 1987)			Galβ3	GalNAcα3	[Fucα2]	Galβ4	GlcNAcβ3	Glcβ	Glcβ	Cer
	H1		4.30	5.03	5.22	4.38	4.63	4.26	4.17	

### Structural characterization of rainbow trout gill subfractions by LC–MS and 1H-NMR

The neutral rainbow trout gill GSL were separated by chromatography on silicic acid and Iatrobeads columns, giving eight subfractions denoted A-1–A-8 (Fig. 4B). The GSL in the gill subfractions were characterized by LC–MS after hydrolysis with rEGCase II (Supplementary Table 1), and fractions A-2 (R_f_:0.34–0.39) and A-5 (R_f_:0.21–0.26) were analyzed by proton NMR for further information on the monosaccharide composition, anomerity and linkage positions of the GSL. These bands were chosen due to that they overlapped with the *A.salmonicida* binding bands (approx. R_f:_ 0.26, 0.27 and 0.38) and contained enough GSL to allow reliable NMR.

### Fraction A-2

**Fig. 5:**
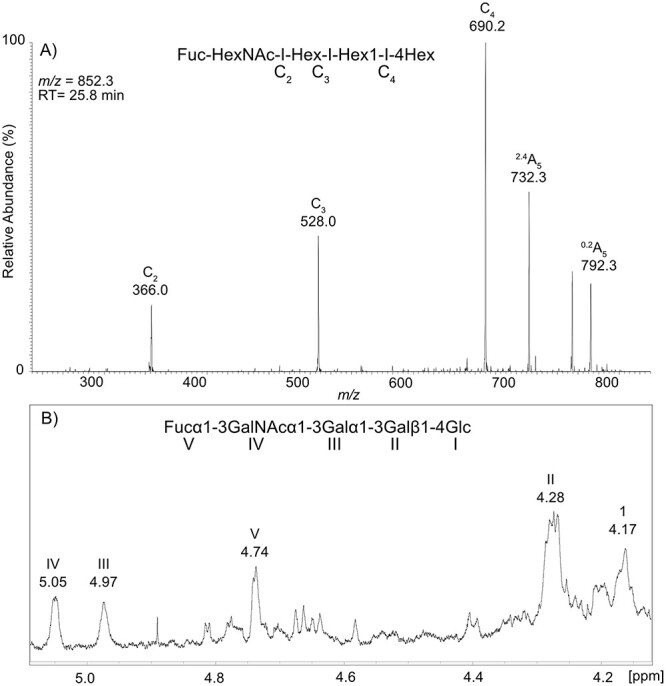
Characterization of fraction A-2 by LC–MS and ^1^H-NMR. A) MS^2^ spectrum of *m/z* 852.3 RT: 25.8 min. B) 700 MHz ^1^H-NMR spectrum displaying the anomeric region of fraction A-2. Panel A shows MS^2^ fragmentation descriptions. See Table 3 for chemical shifts of peaks.

LC–MS of the oligosaccharides derived from fraction A-2 gave a major peak at *m/z* 852. MS/MS of the major peak gave a spectra with C-type fragments at *m/z* 366.0, 528.0 and 690.2, a ^2,4^A fragment at 732.3 and a ^0,2^A fragment at 792.3, indicating oligosaccharides with Fuc-HexNAc-Hex-Hex1-4Hex sequence (Fig. 5A).

^1^H NMR spectra of fraction A-2 (Fig. 5B) showed a mixture of several isoglobo structures. The major structure showed α anomeric H1 peaks at 5.05, 4.97 and 4.74 ppm. These corresponds to GalNAc, Gal and Fuc, respectively. Correlation spectroscopy (COSY) found H2 peaks at 3.97, 3.60 and 3.43, respectively. Major β anomeric H1 peaks were found at ~4.28 and 4.17 corresponding to Gal and Glc, respectively. The MS and NMR data combined suggest that the major GSL is Fucα3GalNAcα3Galα3Galβ4Glcβ1Cer. The fraction is estimated to contain 42% of this structure according to the LC–MS.

### Fraction A-5

**Fig. 6:**
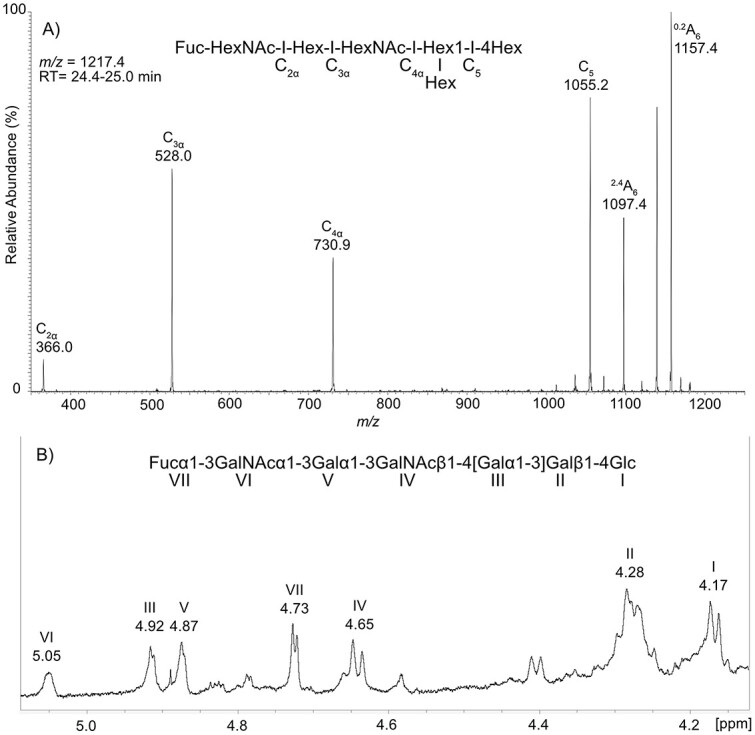
Structural characterization of fraction A-5 by LC–MS and ^1^H-NMR. A) MS^2^ spectrum of *m/z* 1217.4 RT: 24.4–25.0 min. B) 700 MHz ^1^H-NMR spectrum displaying the anomeric region of fraction A-5. Panel A displays MS^2^ fragmentation descriptions. See Table 3 for chemical shifts of peaks.

LC–MS of the oligosaccharides derived from fraction A-5 gave a major peak at *m/z* 1217. MS/MS of the major peak gave a spectra with C-type fragments at *m/z* 366.0, 528.0 and 730.9 and 1055.2, however the *m/z* 1217 peak had no C-type fragment at *m/z* 893. This lack of a C fragment between C_4_α and C_5_ indicated branching. A-type ring-cleavage fragments were found at *m/z* 1097.5 (^2.4^A) and *m/z* 1157.4 (^0,2^A). Thus, the *m/z* 1217 peak structure was denoted as Fuc-HexNAc-Hex-HexNAc-(Hex-)Hex1-4Hex-Cer (Fig. 6A).

^1^H NMR spectra of fraction A-5 (Fig. 6B) showed a mixture of GSL, with major hexa- and hepta-saccharide structures. The major structure showed α anomeric peaks at 5.05, 4.92, 4.88 and 4.73 ppm, corresponding to GalNAc, Gal, Gal and Fuc. COSY further showed interaction with their respective H2 at 3.91, 3.78, 3.74 and 3.45 ppm. The major H1 β anomeric peaks were at 4.65, 4.28 and 4.17 ppm which showed interaction with their H2 at 3.89, 3.45 and 3.05, respectively. This signify GalNAc, Gal and Glc.

The MS and NMR data suggest that the major glycosphingolipid is Fucα3GalNAcα3Galα3GalNAcβ4[Galα3]Galβ4Glcβ1Cer. The fraction is estimated to contain 48% of this structure according to the LC–MS.

## Discussion

Here we isolated and characterized GSLs from rainbow trout skin, gill, esophagus/stomach pyloric caeca and intestine and found that the proportion of the different GSLs differed between the epithelia. Furthermore, *A. salmonicida* bound to neutral GSL from gills and intestine but not to GSL from other epithelia or acidic GSL.

LC–MS in combination with targeted ^1^H-NMR were used for the mapping of the GSLs of the rainbow trout tissues. The LC–MS analysis of the GSL from rainbow trout showed that the majority of GSLs were based on isoglobo- and ganglio core structures, and some were even a hybrid of both. Fucosylated GSL had Fuc-HexNAc-R and HexNAc(Fuc-)HexNAc-R terminals, while sialylated GSL had Neu5Acα2-3 and Neu5Acα2-8. Complex gangliosides (extended GM1 and GD1a) were found in the rainbow trout glycolipid repertoire. These fucosylated types of structures have been found in other fish species such as Pacific salmon (*Oncorhynchus keta*) (Niimura et al. 1999), zebrafish (Yamakawa et al. 2018) and Atlantic salmon (Benktander et al. 2022), but not in mammals (Breimer et al. 1981; Breimer et al. 1982; Johansson et al. 2015; Guo 2022). Sulfated structures were found in the skin, gills, stomach and pyloric caeca, but were absent in the intestine. LC–MS structural assignment of two subfractions, in combination with NMR, identified the fucose terminals as Fucα3GalNAcα3, however some Fucα3GalNAcβ3 terminals are very likely present due to: these structures that has been found in Pacific salmon and English sole (Ostrander et al. 1988; Niimura et al. 1999), isomers of e.g. *m/z* 852 (Fucα3GalNAcα3Galα3Galβ4Glcβ1Cer) were found, and the NMR found minor peaks at 4.66 ppm similar to Niimura et al. (Niimura et al. 1999) for GalNAcβ. The HexNAc(Fuc-)HexNAc-R terminal was not abundant enough to allow examination in detail.

The ganglio−/isoglobo- hybrid GSL found in gill and stomach were similar to the gangliosides identified, with the Neu5Acα2-3 exchanged for a Galα1-3.

Similar types of GSL structures were also identified in Atlantic salmon even though the terminal fucose linkage was not defined (Benktander et al. 2022). In zebrafish many similar structures are present, with the exception that no ganglio/isoblobo hybrid structures and only Fucα3GalNAcβ were found, and no α-linked GalNAc was identified (Yamakawa et al. 2018). A study on rainbow trout liver GSL also demonstrated some overlapping compounds, and as well as acetyl-GD3 (Ostrander et al. 1991), which was not found in this study. The acetyl group is sensitive to the alkaline methanolysis of the isolation method used, thus this GSL would not be detected due to conversion into GD3 if present.

Compared to the Atlantic salmon GSL (Benktander et al. 2022) the rainbow trout had a higher percentage of terminal fucose in all examined epithelia. Otherwise, the terminal moieties of skin and gill GSL were quite similar between the two species. However, the rainbow trout stomach had less sialylation. The rainbow trout pyloric caeca GSL were dominated by fucose and sialic acids, while the Atlantic salmon had more terminal hexoses. In the intestine, the rainbow trout GSL had less sialylation but more fucosylation compared to the Atlantic salmon GSL.

Microbial adhesion to GSL provide an intimate association with the host, and a large variety of bacterial pathogens carry adhesins with specific epitopes that bind to GSL (Aspholm-Hurtig et al. 2004; Benktander et al. 2012; Benktander et al. 2013; Padra et al. 2018). Adhesion to GSLs is often essential for pathogens in order to infect the host. An example of this would be *Helicobacter pylori* that have the adhesin BabA that binds the fucosylated blood group structures (Borén et al. 1993; Benktander et al. 2012). After prolonged infection, *H. pylori* switch to relying on the SabA adhesin, which binds to the acidic saccharide structures that become more prevalent in the stomach due to infection induced inflammation (Mahdavi et al. 2002; Aberg et al. 2014; Benktander et al. 2018). Here we showed that *A. salmonicida* bound to neutral GSL from the gill, while no binding to acidic GSL was detected. Still *A. salmonicida* has been found to bind to sialic acid containing epitopes on mucins (Padra et al. 2014; Padra et al. 2017). However, we recently found that *A. salmonicida* only binds to Neu5Acα2-6 while Neu5Acα2-3 is not recognized, at least when these epitopes are present in the form of sialolactose glycoconjugates (Benktander et al. 2022). Neu5Acα2–6 is present on Atlantic salmon mucins, but we found no α2,6-linked Neu5Ac in the GSL repertoires of either Atlantic salmon or rainbow trout in any of the tissues examined (Benktander et al. 2019; Benktander et al. 2022).

After separation of the neutral GSL from the gill into subfractions and LC–MS in combination with ^1^H-NMR, two GSL were identified: Fucα3GalNAcα3Galα3Galβ4Glcβ1Cer and Fucα3GalNAcα3Galα3GalNAcβ4(Galα3)Galβ4Glcβ1Cer. A common theme to these structures is the Fucα3GalNAc terminal, which possibly could be a binding motif for *A. salmonicida*. Since binding also was obtained in the tetra-penta glycosylceramide area among the intestinal GSLs, it could be speculated that *A. salmonicida* could bind some terminal GalNAc, with or without fucosylation. However, the *A. salmonicida* binding specificity needs further investigation, and the only claim we make is that we detected binding to netutral GSL from gill and intestine. The fact that *A. salmonicida* binding GSL provide close interactions with the epithelial cells of the rainbow trout gills and intestine, is in line with findings that the gills and fins are the sites the pathogen first colonizes (Bartkova et al. 2017), and that *A. salmonicida* can utilize the intestine as a translocation route (Jutfelt et al. 2006; Jutfelt et al. 2008).

In conclusion, epitopes on the rainbow trout GSL glycans differed between epithelial sites but had both similarities and differences with the Atlantic salmon GSL epitopes from the same epithelial site. These differences were sufficiently pronounced to lead to differences in pathogen binding. The identification of the GSL epitopes in gill epithelia, that provide intimate *A. salmonicida* interaction and where the bacteria also can be found hours after exposure (Bartkova et al. 2017), is a step towards understanding *A. salmonicida* infection mechanisms and potential manners to interfere in this process for protecting against infections in aquaculture.

## Materials and methods

### Fish, sampling and ethics

Rainbow trout (300–500 g) were purchased from Vänneåns fish farm and were maintained in concrete tanks (1 m^3^) supplied with 10 °C freshwater from a recirculating aquaculture system. Fish were fed a commercial diet 3 times per week. Fish were quickly netted and euthanized in freshwater containing 200 mg/L ethyl 3-aminobenzoate methanesulfonate salt (MS-222; Sigma-Aldrich, Germany) buffered with Na_2_CO_3_. Skin, gills, stomach, pyloric caeca and intestine were collected and each tissue pooled from 3 fish and frozen to −80 °C until analysis. The skin was collected from the sides of the fish, by pulling the skin away from the muscles. The gills collected were cut from the gill arches. In stomach and intestine, the chyme and fecal contents were removed before collecting the samples. All experimental procedures were performed and approved by the Gothenburg ethics committee for animal testing (permit nr. 5.8.18-15096/2018).

### GSL isolation

The isolation was done as described previously (Karlsson 1987). Pooled tissue samples from three fish tissues from 300–500 g rainbow trout were taken from the −80 °C freezer and lyophilized (See Table 1). The dried samples were extracted by running 24 h in an Soxleth apparatus with boiling chloroform: methanol solution (2:1 ratio), then another 24 h in chloroform: methanol solution (1:9 ratio). The solutions were dried and pooled. By adding 10 mL/g of 0.2 M KOH in methanol, for a mild alkaline methanolysis the ester linkages of glycerol-based phospholipids were hydrolyzed. Note that eventual ester linkage modifications and other non-alkaline stable modifications on the GSL may have been lost in this step. The solution was dialysed against running tap water for at least 3 days by adding chloroform and dialysed water to get a two-phase system in molecular membrane tubing (MWCO:12–14 kD, Spectra/Por). The dialysed solution was dried and then subjected to a silicic acid column to separate the fatty acids and other unpolar compounds from the GSL. Next a column with diethylaminoethyl (DEAE) ion exchange resin was used to separate the GSL with acidic moieties from the neutral. The acidic fractions eluted with 5% LiCl in methanol were dialyzed and dried again. The neutral fractions were acetylated with a 1:1:1 mixture of chloroform: pyridine: acetic anhydride. After addition of methanol the fraction was dried. The acetylated GSLs was separated from the sphingomyelin based on polarity by using a silicic acid column. Thereafter the fractions were deacetylated again by adding 0.2 M KOH in methanol, followed by dialysis. The dried fractions were chromatographed on a second DEAE ion exchange column (the dialyzed acid fraction were pooled with the acid fraction from first DEAE), then ran on a final silicic acid column to further separate away any remaining unpolar compounds.

The neutral GSL from rainbow trout gills were further separated by silicic acid chromatography. First, GSLs with one and two monosaccharides were separated for the GSL with longer saccharide chains by eluting them with 5%–30% methanol:chloroform (by volume) and then eluting the rest in 75% and 100% methanol:chloroform (by volume). Then the fraction with complex GSL was further separated on an Iatrobeads column (Iatron Labs., Tokyo, Japan) eluted with 60:35:8 chloroform:methanol:water (by volume). The fractions were pooled according to their migration on thin-layer chromatograms. This gave eight fractions called A-1 to A-8.

### Liquid chromatography–mass spectrometry (LC–MS)

Preparation of the neutral GSLs for LC–MS was done as previously described (Karlsson et al. 2010). Neutral GSLs were examined after rEGCase II digestion to facilitate the study of the glycan part on LC–MS using a pourous graphitized carbon column, while the acid GSLs were ran on LC–MS in native form with columns packed with the hydrophilic interaction liquid chromatography (HILIC) material polyamine II (YMC). Both the GSL derived neutral oligosaccharides and the native acidic GSLs were analyzed in negative-ion mode using an LTQ mass spectrometer (Thermo Scientific) as previously described (Jin et al. 2015), with the following conditions: electrospray voltage: 3.5 kV; capillary voltage: −33.0 V; capillary temperature: 300 °C; sheath gas: compressed air. For the LC–MS; full scans were performed in the mass range *m*/*z* 380–2000. MS/MS; minimal signal 300 counts; normalized collisional energy 35%; activation time 30 ms; isolation width *m*/*z* 2.0. The Xcalibur software (version 2.0.7, Thermo Scientific) was used for data analysis, glycans were manually annotated from their MS/MS spectra and, when possible, validated by the Unicarb-DB (Hayes et al. 2011) database. The tentative structures were denoted by using B, C, X and Y fragments from the MS/MS to find monosaccharide mass while ring-cleavage fragments such as 2.4A, 0,2A 0.2X and 0,4A fragments to find linkage and potential branching of the monosaccharide chain. When calculating the relative abundances of the total glycans, neutral and acidic glycans were combined based on the weight of the neutral and acidic GSL fractions.

### Immunofluorescence

Paraffin embeded sections from the rainbow tissues were deparaffinized and rehydrated. For antigen retrieval, sections were heated in 0.01 M citric acid buffer pH 6 at 99 °C for 10 min. Non-specific binding was blocked with 5% bovine serum diluted in phosphate-buffered saline (PBS), pH 7.3, and sections were incubated with an antibody recognizing sulfatides (MAB1326, monoclonal mouse anti-O4, R&D systems) diluted (1:100) in blocking buffer at 4 °C O/N. The following day, slides were rinsed in PBS containing Tween 20 (0.05%) and incubated with Alexa Flour 488-conjugated goat anti-mouse IgG (ab150113, Abcam) diluted (1:1500) in blocking buffer at RT for 1 h. Subsequently, slides were rinsed in PBS and incubated with CellMask (C10046, Thermo Fisher Scientific) diluted 1:14000 in PBS at RT for 30 min to outline the tissue. Finally sections were rinsed in PBS and mounted with DAPI containing prolong Gold anti-fade reagent (P36935, Thermo Fisher Scientific). Images were captured with an Eclipse 90i fluorescence microscope (Nikon).

### NMR

^1^H NMR were run on a Brucker 700-MHz spectrometer at 30 °C. Samples were subjected to a deuterium exchange and dissolved in dimethyl sulfoxide/D_2_O (98:2 by volume).

The NMR data were interpretated using data from reference GSL in combination with results from LC–MS.

### Bacteria culture conditions

*A. salmonicida* subspecies *salmonicida* strain VI-88/09/03175 (culture collection, Central Veterinary Laboratory, Oslo, Norway) was cultured at 16 °C on tryptic soy agar plates for 48 h after being brought up from −80 °C. *A. salmonicida* were radiolabeled by the addition of 50 μCi ^35^S-methionine (PerkinElmer; NEG77207MC) diluted in 0.5 mL PBS, that were added to the culture plates. After incubation for 16–24 h at respective culturing conditions, the bacteria were harvested, centrifuged three times and suspended to 1 × 10^8^ CFU/mL in PBS. The specific activity of the suspensions were approximately 1 cpm per 100 bacteria.

### Chromatogram binding assay

Thin-layer chromatograms were made by running the GSL (80 μg of the unfractionated GSL and 8 μg GSL for the isolated fractions) in a chloroform:methanol:water mixture in the proportions 60:35:8 (by volume). The dried chromatograms were dipped for 1 min in diethylether/*n*-hexane (1:5, by volume) containing 0.5% (w/v) polyisobutylmethacrylate (Sigma-Aldrich; 181544). After drying, the chromatograms were soaked in PBS/BSA (PBS containing 2% bovine serum albumin (w/v), 0.1% NaN_3_ (w/v) and 0.1% Tween 20 (by volume)) for 2 h at room temperature. The chromatograms were then covered with radiolabeled bacteria diluted in PBS/BSA (2–5 × 10^6^ cpm/mL). Incubation was done for 2 h at room temperature, followed by repeated washings with PBS. The chromatograms were thereafter exposed to XAR-5 X-ray films (Carestream; 8941114) for 12–48 h. Due to differences in migration for aluminum- and glass-backed plates and air humidity, some small differences in R_f_ values might occur between thin-layer chromatograms.

## Supplementary Material

Supplementary_table_I_cwae055

## Data Availability

Raw mass spectrometry data is available at http://doi.org/10.50821/GLYCOPOST-GPST000221.
