# Supplementary material for: Characterization of the rainbow trout (Oncorhynchus mykiss) mucosal glycosphingolipid repertoire and Aeromonas salmonicida binding to neutral glycosphingolipids
Source: Glycobiology. 2024 Aug 7;34(9):cwae055. doi: 10.1093/glycob/cwae055 (PMC11303275; doi:10.1093/glycob/cwae055)
Supplement: Supplementary_table_I_cwae055 [file supplementary_table_i_cwae055.docx]

| **Neutral GSL** |  | | | | | |  | |  | |  | |  |  |  |  |  |
| --- | --- | --- | --- | --- | --- | --- | --- | --- | --- | --- | --- | --- | --- | --- | --- | --- | --- |
|  |  | | | | | |  | |  | |  | |  |  |  |  |  |
| [M-H+]- | Tentative structure | | | | | |  | |  | | Common name | | Skin | Gills | Stomach | Pyloric caeca | Intestine |
| 706.2 | GalNAcβ1-3Galα1-4Galβ1-4Glc | | | | | |  | |  | | Gb4 | | 0.0 | 5.5 | 3.4 | 0.0 | 11.9 |
| 706.2 | Galß1-4GlcNAcß1-4Galβ1-4Glc | | | | | |  | |  | | nLc4 | | 3.6 | 0.0 | 1.1 | 0.0 | 2.5 |
| 852.3 | Fucα1-3GalNAcα1-3Galα1-3Galβ1-4Glc | | | | | |  | |  | | Fucα1-3GalNAcα1-3iGb3 | | 0.0 | 36.0 | 0.0 | 0.0 | 0.0 |
| 852.3 | Fucα1-3GalNAcβ1-3Galα1-3Galβ1-4Glc | | | | | |  | |  | | Fucα1-3iGb4 | | 0.0 | 6.2 | 0.0 | 0.0 | 0.0 |
| 868.2 | Galβ1-3GalNAcβ1-4(Galα1-3)Galβ1-4Glc | | | | | |  | |  | | Gg/iGb5 | | 0.0 | 1.0 | 17.9 | 0.0 | 0.0 |
| 868.2 | Galα1-3Galß1-4GlcNAcß1-3Galβ1-4Glc | | | | | |  | |  | | Galα1-3nLc4 | | 61.3 | 2.2 | 14.3 | 0.0 | 12.7 |
| 909.2 | GalNAc1-3Galß1-3GalNAcß1-4Galβ1-4Glc | | | | | |  | |  | | GalNAc-Gg4 | | 0.0 | 0.0 | 2.6 | 0.0 | 0.0 |
| 1014.2 | Fucα1-3GalNAc1-3Galα1-4(Galα1-3)Galβ1-4Glc | | | | | |  | |  | | Fucα1-3GalNAc-Gb/iGb4 | | 0.0 | 3.2 | 0.0 | 0.0 | 0.0 |
| 1055.3 | Fucα1-3GalNAc1-3Galß1-3GalNAcß1-4Galβ1-4Glc | | | | | |  | |  | | Fucα1-3GalNAc1-3Gg4 | | 35.1 | 31.9 | 32.9 | 83.3 | 70.8 |
| 1071.3 | Gal-HexAc1-3Galß1-4GlcNAcβ1-3Galβ1-4Glc | | | | | |  | |  | | nLc6 | | 0.0 | 0.0 | 0.7 | 16.7 | 2.1 |
| 1112.3 | HexNAc-HexNAc1-3Galß1-3GalNAcß1-4Galβ1-4Glc | | | | | |  | |  | | HexNAc-Gg5 | | 0.0 | 0.0 | 8.3 | 0.0 | 0.0 |
| 1217.3 | Fucα1-3GalNAcα1-3Galα1-3GlcNAcβ1-4(Galα1-3)Galβ1-4Glc | | | | | |  | |  | | Fucα1-3GalNAcα1-3Gg/iGb5 | | 0.0 | 14.0 | 18.8 | 0.0 | 0.0 |
|  |  | | | | | |  | |  | |  | |  |  |  |  |  |
| **Acid GSL** |  | | | | | |  | |  | |  | |  |  |  |  |  |
|  |  | | | | | | Ceramide | |  | |  | |  |  |  |  |  |
| [M-H+]- | Supposed structure | | | | | | Sphingosine type | | Fatty acid | | Common name | | Skin | Gills | Stomach | Pyloric caeca | Intestine |
| 2283.4 | Galß1-3GalNAcß1-4Galß1-3GalNAcß1-4(NeuAcα2-8NeuAcα2-3)Galß1-4Glcß1-Cer | | | | | | d18:1 | | 24:1 | | 2NeuAc-hexa | | 0.2 | 0.0 | 0.8 | 0.0 | 0.0 |
| 2301.4 | Galß1-3GalNAcß1-4Galß1-3GalNAcß1-4(NeuAcα2-8NeuAcα2-3)Galß1-4Glcß1-Cer | | | | | | d18:1 | | h24:0 | | 2NeuAc-hexa | | 0.0 | 0.0 | 0.2 | 0.0 | 0.0 |
| 2145.1 | Fucα1-3GalNAc1-3Galß1-3GalNAcß1-4(NeuAcα2-8NeuAcα2-3)Galß1-4Glcß1-Cer | | | | | | d18:1 | | h14:0 | | Fuc-GalNAc-GD1b | | 0.0 | 0.0 | 0.0 | 3.8 | 5.0 |
| 2283.4 | Fucα1-3GalNAc1-3Galß1-3GalNAcß1-4(NeuAcα2-8NeuAcα2-3)Galß1-4Glcß1-Cer | | | | | | d18:1 | | h24:1 | | Fuc-GalNAc-GD1b | | 0.0 | 0.0 | 0.0 | 33.0 | 15.6 |
| 2301.2 | Fucα1-3GalNAc1-3Galß1-3GalNAcß1-4(NeuAcα2-8NeuAcα2-3)Galß1-4Glcß1-Cer | | | | | | t18:0 | | h24:1 | | Fuc-GalNAc-GD1b | | 0.0 | 0.0 | 0.0 | 16.8 | 9.6 |
| 2299.2 | Fucα1-3GalNAc1-3Galß1-3GalNAcß1-4(NeuGcα2-8NeuAcα2-3)Galß1-4Glcß1-Cer | | | | | | d18:1 | | h24:1 | | Fuc-GalNAc-GD1b (NeuAc+NeuGc) | | 0.0 | 0.0 | 0.0 | 0.0 | 6.8 |
| 1855.9 | Fucα1-3GalNAc1-3Galß1-3GalNAcß1-4(NeuAcα2-3)Galß1-4Glcß1-Cer | | | | | | t18:0 | | 14:0 | | Fuc-GalNAc-GM1 | | 0.0 | 2.2 | 0.0 | 0.0 | 0.0 |
| 1884 | Fucα1-3GalNAc1-3Galß1-3GalNAcß1-4(NeuAcα2-3)Galß1-4Glcß1-Cer | | | | | | t18:0 | | 16:0 | | Fuc-GalNAc-GM1 | | 0.0 | 2.1 | 0.2 | 0.0 | 0.0 |
| 1976 | Fucα1-3GalNAc1-3Galß1-3GalNAcß1-4(NeuAcα2-3)Galß1-4Glcß1-Cer | | | | | | d18:1 | | 24:1 | | Fuc-GalNAc-GM1 | | 0.0 | 1.8 | 1.0 | 0.0 | 0.0 |
| 1992.1 | Fucα1-3GalNAc1-3Galß1-3GalNAcß1-4(NeuAcα2-3)Galß1-4Glcß1-Cer | | | | | | d18:1 | | h24:1 | | Fuc-GalNAc-GM1 | | 0.0 | 0.0 | 0.1 | 7.1 | 8.3 |
| 1994.1 | Fucα1-3GalNAc1-3Galß1-3GalNAcß1-4(NeuAcα2-3)Galß1-4Glcß1-Cer | | | | | | d18:1 | | h24:0 | | Fuc-GalNAc-GM1 | | 0.0 | 4.2 | 1.2 | 4.4 | 0.0 |
| 2008.1 | Fucα1-3GalNAc1-3Galß1-3GalNAcß1-4(NeuAcα2-3)Galß1-4Glcß1-Cer | | | | | | d18:1 | | h25:1 | | Fuc-GalNAc-GM1 | | 0.0 | 0.3 | 0.0 | 0.0 | 6.0 |
| 2010.1 | Fucα1-3GalNAc1-3Galß1-3GalNAcß1-4(NeuAcα2-3)Galß1-4Glcß1-Cer | | | | | | t18:0 | | h24:1 | | Fuc-GalNAc-GM1 | | 0.0 | 0.6 | 0.0 | 7.2 | 6.9 |
| 1918 | NeuAcα2-3Galß1-3GalNAcß1-4(NeuAcα2-3)Galß1-4Glcß1-Cer | | | | | | d18:1 | | 24:1 | | GD1a | | 1.4 | 30.2 | 2.9 | 0.0 | 0.0 |
| 1934 | NeuAcα2-3Galß1-3GalNAcß1-4(NeuAcα2-3)Galß1-4Glcß1-Cer | | | | | | d18:1 | | h24:1 | | GD1a | | 0.6 | 0.0 | 0.4 | 0.0 | 0.0 |
| 1936 | NeuAcα2-3Galß1-3GalNAcß1-4(NeuAcα2-3)Galß1-4Glcß1-Cer | | | | | | d18:1 | | h24:0 | | GD1a | | 0.0 | 13.2 | 0.9 | 0.0 | 0.0 |
| 1918 | Galß1-3GalNAcß1-4(NeuAcα2-8NeuAcα2-3)Galß1-4Glcß1-Cer | | | | | | d18:1 | | 24:1 | | GD1b | | 1.2 | 0.0 | 1.9 | 0.0 | 0.0 |
| 1934 | Galß1-3GalNAcß1-4(NeuAcα2-8NeuAcα2-3)Galß1-4Glcß1-Cer | | | | | | d18:1 | | h24:1 | | GD1b | | 0.3 | 0.0 | 0.3 | 0.0 | 4.6 |
| 1936 | Galß1-3GalNAcß1-4(NeuAcα2-8NeuAcα2-3)Galß1-4Glcß1-Cer | | | | | | d18:1 | | h24:0 | | GD1b | | 1.5 | 0.0 | 0.6 | 0.0 | 0.0 |
| 1415.7 | NeuAcα2-8NeuAcα2-3Galß1-4Glcß1-Cer | | | | | | d18:1 | | 14:0 | | GD3 | | 2.6 | 0.0 | 0.9 | 0.0 | 0.0 |
| 1442.7 | NeuAcα2-8NeuAcα2-3Galß1-4Glcß1-Cer | | | | | | d18:1 | | 16:0 | | GD3 | | 4.0 | 0.0 | 0.6 | 0.0 | 0.0 |
| 1458.7 | NeuAcα2-8NeuAcα2-3Galß1-4Glcß1-Cer | | | | | | d18:1 | | h16:0 | | GD3 | | 0.0 | 0.0 | 0.0 | 2.2 | 0.0 |
| 1552.8 | NeuAcα2-8NeuAcα2-3Galß1-4Glcß1-Cer | | | | | | d18:1 | | 24:1 | | GD3 | | 7.7 | 2.3 | 2.3 | 3.2 | 0.0 |
| 1568.8 | NeuAcα2-8NeuAcα2-3Galß1-4Glcß1-Cer | | | | | | d18:1 | | h24:1 | | GD3 | | 2.3 | 0.0 | 1.8 | 1.8 | 1.8 |
| 1570.8 | NeuAcα2-8NeuAcα2-3Galß1-4Glcß1-Cer | | | | | | d18:1 | | h24:0 | | GD3 | | 2.5 | 2.9 | 3.0 | 2.5 | 1.7 |
| 1123.7 | NeuAcα2-3Galß1-4Glcß1-Cer | | | | | | d18:1 | | 14:0 | | GM3 | | 0.4 | 0.2 | 0.3 | 0.0 | 0.0 |
| 1151.7 | NeuAcα2-3Galß1-4Glcß1-Cer | | | | | | d18:1 | | 16:0 | | GM3 | | 0.4 | 0.2 | 0.3 | 0.0 | 0.0 |
| 1261.8 | NeuAcα2-3Galß1-4Glcß1-Cer | | | | | | d18:1 | | 24:1 | | GM3 | | 3.7 | 1.8 | 0.7 | 0.9 | 0.0 |
| 1277.8 | NeuAcα2-3Galß1-4Glcß1-Cer | | | | | | d18:1 | | h24:1 | | GM3 | | 1.6 | 1.3 | 0.4 | 0.4 | 0.0 |
| 1279.8 | NeuAcα2-3Galß1-4Glcß1-Cer | | | | | | d18:1 | | h24:0 | | GM3 | | 1.6 | 1.6 | 0.4 | 0.6 | 0.0 |
| 977.7 | NeuAcα2-3Galß1-Cer | | | | | | d18:1 | | h14:0 | | GM4 | | 0.0 | 0.0 | 0.0 | 0.0 | 4.8 |
| 1005.8 | NeuAcα2-3Galß1-Cer | | | | | | d18:1 | | h16:0 | | GM4 | | 0.0 | 0.0 | 0.0 | 0.0 | 0.7 |
| 1115.8 | NeuAcα2-3Galß1-Cer | | | | | | d18:1 | | h24:1 | | GM4 | | 0.2 | 0.0 | 0.0 | 6.0 | 22.3 |
| 1129.8 | NeuAcα2-3Galß1-Cer | | | | | | d18:1 | | h25:1 | | GM4 | | 0.0 | 0.0 | 0.0 | 0.6 | 0.8 |
| 1131.8 | NeuAcα2-3Galß1-Cer | | | | | | d18:1 | | h25:0 | | GM4 | | 0.0 | 0.0 | 0.0 | 0.6 | 1.3 |
| 2209.1 | NeuAcα2-3Galß1-3GalNAcß1-4(NeuAcα2-8NeuAcα2-3)Galß1-4Glcß1-Cer | | | | | | d18:1 | | 24:1 | | GT1b | | 1.0 | 2.9 | 3.3 | 0.0 | 0.0 |
| 2059.1 | HexNAc-(Fuc-)HexNAc1-3Galß1-3GalNAcß1-4(NeuAcα2-3)Galß1-4Glcß1-Cer | | | | | | t18:0 | | 14:0 | | HexNAc-(Fuc-)HexNAc-GM1 | | 0.0 | 1.0 | 0.0 | 0.0 | 0.0 |
| 2197.1 | HexNAc-(Fuc-)HexNAc1-3Galß1-3GalNAcß1-4(NeuAcα2-3)Galß1-4Glcß1-Cer | | | | | | d18:1 | | h24:0 | | HexNAc-(Fuc-)HexNAc-GM1 | | 0.0 | 4.0 | 0.6 | 0.0 | 0.0 |
| 1853.9 | NeuAcα2-3Galß1-3GalNAcß1-4Galß1-3GalNAcß1-4Galß1-4Glcß1-Cer | | | | | | d18:1 | | 14:0 | | NeuAc-hexa | | 0.0 | 0.0 | 0.0 | 1.7 | 4.0 |
| 750.8 | SO3-Galß1-Cer | | | | | | d18:1 | | 14:0 | | Sulfatide | | 2.8 | 1.3 | 1.5 | 1.1 | 0.0 |
| 888.8 | SO3-Galß1-Cer | | | | | | d18:1 | | 24:1 | | Sulfatide | | 50.9 | 19.5 | 37.7 | 6.1 | 0.0 |
|  |  | | | | | |  | |  | |  | |  |  |  |  |  |
|  |  | | | | | |  | |  | | Summary (acid GSL): | | Skin | Gills | Stomach | Pyloric caeca | Intestine |
|  |  | | | | | |  | |  | | Sulfatide | | 66.8 | 26.9 | 74.7 | 7.3 | 0.0 |
|  |  | | | | | |  | |  | | GM4 | | 0.2 | 0.0 | 0.0 | 7.2 | 29.9 |
|  |  | | | | | |  | |  | | GM3 | | 7.7 | 5.1 | 2.0 | 1.9 | 0.0 |
|  |  | | | | | |  | |  | | GD3 | | 19.1 | 5.2 | 8.6 | 9.6 | 3.4 |
|  |  | | | | | |  | |  | | GD1a | | 2.0 | 43.4 | 4.3 | 0.0 | 0.0 |
|  |  | | | | | |  | |  | | GD1b | | 3.0 | 0.0 | 2.8 | 0.0 | 4.6 |
|  |  | | | | | |  | |  | | GT1b | | 1.0 | 2.9 | 3.3 | 0.0 | 0.0 |
|  |  | | | | | |  | |  | | NeuAc-Hexa | | 0.0 | 0.0 | 0.0 | 1.7 | 4.0 |
|  |  | | | | | |  | |  | | Fuc-GalNAc-GM1 | | 0.0 | 11.3 | 2.5 | 18.7 | 21.2 |
|  |  | | | | | |  | |  | | Fuc-GalNAc-GD1b | | 0.0 | 0.0 | 0.0 | 53.7 | 30.1 |
|  |  | | | | | |  | |  | | Fuc-GalNAc-GD1b (NeuAc+NeuGc) | | 0.0 | 0.0 | 0.0 | 0.0 | 6.8 |
|  |  | | | | | |  | |  | |  | |  |  |  |  |  |
|  | |  |  |  |  |  | |  | |  | |  |  |  |  |  |  |
| [M-H+]- | Supposed structure | | | | | | A-1 | | A-2 | | A-3 | | A-4 | A-5 | A-6 | A-7 | A-8 |
| 503.2 | Galα1-3Galß1-4Glc | | | | | | 100.0 | | 1.4 | | 0.0 | | 0.0 | 0.0 | 0.0 | 0.0 | 0.0 |
| 706.3 | GalNAcβ1-4(Galα1-3)Galß1-4Glc | | | | | | 0.0 | | 7.2 | | 0.7 | | 0.4 | 0.0 | 0.0 | 0.0 | 0.0 |
| 706.3 | Galß1-3GalNAcß1-4Galß1-4Glc | | | | | | 0.0 | | 0.0 | | 3.1 | | 1.8 | 0.0 | 0.0 | 0.0 | 0.0 |
| 706.3 | GalNAcß1-3Galα1-3Galß1-4Glc | | | | | | 0.0 | | 2.0 | | 0.0 | | 0.0 | 0.0 | 0.0 | 0.0 | 0.0 |
| 706.3 | GalNAcß1-3Galα1-4Galß1-4Glc | | | | | | 0.0 | | 0.7 | | 0.0 | | 0.0 | 0.0 | 0.0 | 0.0 | 0.0 |
| 852.3 | Fucα1-3GalNAcα1-3Galα1-3Galß1-4Glc | | | | | | 0.0 | | 41.9 | | 24.4 | | 5.7 | 1.8 | 2.0 | 2.3 | 3.9 |
| 852.3 | Fucα1-3GalNAcβ1-4(Galα1-3)Galß1-4Glc | | | | | | 0.0 | | 19.3 | | 10.4 | | 0.0 | 0.0 | 0.0 | 0.0 | 0.0 |
| 852.3 | Fucα1-3GalNAcβ1-3Galα1-3Galß1-4Glc | | | | | | 0.0 | | 21.1 | | 3.1 | | 0.8 | 0.3 | 0.3 | 0.2 | 0.4 |
| 868.3 | Galβ1-3GalNAcß1-4(Galα1-3)Galß1-4Glc | | | | | | 0.0 | | 0.0 | | 5.7 | | 6.5 | 0.4 | 0.0 | 0.0 | 0.0 |
| 868.3 | Galα1-3Galβ1-4GlcNAcβ1-3Galß1-4Glc | | | | | | 0.0 | | 1.2 | | 12.7 | | 5.3 | 0.6 | 0.0 | 0.0 | 0.0 |
| 1014.3 | Fucα1-3GalNAc1-3Galα1-3(Galα1-4)Galß1-4Glc | | | | | | 0.0 | | 0.4 | | 14.4 | | 14.4 | 1.0 | 0.6 | 0.5 | 1.1 |
| 1055.3 | Fucα1-3GalNAc1-3Galα1-3(GalNAcβ1-4)Galß1-4Glc | | | | | | 0.0 | | 0.0 | | 2.5 | | 14.9 | 2.3 | 0.0 | 0.0 | 0.0 |
| 1055.3 | Fucα1-3GalNAc1-3Galβ1-3GalNAcβ1-4Galß1-4Glc | | | | | | 0.0 | | 0.7 | | 7.2 | | 18.8 | 12.5 | 4.3 | 3.7 | 2.5 |
| 1055.3 | Fucα1-3GalNAc1-3Galβ1-3GalNAcβ1-4Galß1-4Glc | | | | | | 0.0 | | 0.9 | | 9.6 | | 23.3 | 23.1 | 19.4 | 26.6 | 14.3 |
| 1055.3 | HexNAc-(Fuc-)HexNAc1-3Galα-3Galß1-4Glc | | | | | | 0.0 | | 3.0 | | 6.0 | | 1.7 | 0.0 | 0.0 | 0.0 | 0.0 |
| 1055.3 | Fucα1-3GalNAc1-3Galβ1-4GlcNAcβ1-3Galß1-4Glc | | | | | | 0.0 | | 0.0 | | 0.0 | | 1.5 | 0.0 | 0.0 | 0.0 | 0.0 |
| 1071.3 | HexNAc-Galß1-3GalNAcß1-3Galα1-3Galß1-4Glc | | | | | | 0.0 | | 0.0 | | 0.0 | | 1.3 | 1.0 | 0.2 | 0.0 | 0.0 |
| 1071.3 | Gal-HexNAc1-3Galβ1-4GlcNAcβ1-3Galß1-4Glc | | | | | | 0.0 | | 0.0 | | 0.0 | | 0.5 | 1.0 | 0.4 | 0.0 | 0.0 |
| 1217.3 | Fucα1-3GalNAcα1-3Galα1-3GalNAcβ1-4(Galα1-3)Galß1-4Glc | | | | | | 0.0 | | 0.0 | | 0.1 | | 2.0 | 47.7 | 37.2 | 20.8 | 9.5 |
| 1258.3 | HexNAc-(Fuc-)HexNAc-Galβ1-3GalNAcβ1-4Galß1-4Glc | | | | | | 0.0 | | 0.0 | | 0.0 | | 1.0 | 5.0 | 19.1 | 24.1 | 42.2 |
| 1258.3 | HexNAc-(Fuc-)HexNAc-Galβ1-3GalNAcβ1-4Galß1-4Glc | | | | | | 0.0 | | 0.0 | | 0.0 | | 0.3 | 2.1 | 3.6 | 0.0 | 0.0 |
| 1420.4 | HexNAc-(Fuc-)HexNAc-Galβ1-3GalNAcβ1-4(Galα1-3)Galß1-4Glc | | | | | | 0.0 | | 0.0 | | 0.0 | | 0.0 | 1.3 | 12.9 | 17.9 | 8.9 |
| 1566.4 | Fucα1-3GalNAc1-3Gal-(Fuc-)HexNAcβ1-3Gal-GlcNAc1-3Galß1-4Glc | | | | | | 0.0 | | 0.0 | | 0.0 | | 0.0 | 0.0 | 0.0 | 0.8 | 1.9 |
| 1566.4 | Fuc{ Fuc-HexNAc-Gal-HexNAcβ1-3Gal-GlcNAc1-3Galß1-4Glc | | | | | | 0.0 | | 0.0 | | 0.0 | | 0.0 | 0.0 | 0.0 | 1.3 | 1.9 |
| 1566.4 | Fuc{ HexNAc-Gal-HexNAcβ1-3Gal-(Fuc-)HexNAc1-3Galß1-4Glc | | | | | | 0.0 | | 0.0 | | 0.0 | | 0.0 | 0.0 | 0.0 | 1.4 | 5.4 |
| 1623.3 | HexNAc-(Fuc-)HexNAc-Gal-HexNAc1-3Galβ1-3GalNAcß1-4Galß1-4Glc | | | | | | 0.0 | | 0.0 | | 0.0 | | 0.0 | 0.0 | 0.0 | 0.4 | 3.3 |
| 1728.6 | Gal-(Fuc-)HexNAc-Gal-(Fuc-)HexNAc1-3Galβ1-3GalNAcß1-4Galß1-4Glc | | | | | | 0.0 | | 0.0 | | 0.0 | | 0.0 | 0.0 | 0.0 | 0.0 | 3.8 |
| 1770.7 | HexNAc-(Fuc-)HexNAc-Gal-(Fuc-)HexNAc1-3Galβ1-3GalNAcß1-4Galß1-4Glc | | | | | | 0.0 | | 0.0 | | 0.0 | | 0.0 | 0.0 | 0.0 | 0.0 | 1.0 |

**Supplementary table I**
